# Adaptive Multi-ROI Agricultural Robot Navigation Line Extraction Based on Image Semantic Segmentation

**DOI:** 10.3390/s22207707

**Published:** 2022-10-11

**Authors:** Xia Li, Junhao Su, Zhenchao Yue, Fangtao Duan

**Affiliations:** 1 Tianjin Key Laboratory for Advanced Mechatronic System Design and Intelligent Control, School of Mechanical Engineering, Tianjin University of Technology, Tianjin 300384, China; 2 National Demonstration Center for Experimental Mechanical and Electrical Engineering Education, Tianjin University of Technology, Tianjin 300384, China

**Keywords:** semantic segmentation, transfer learning, navigation, image processing, path recognition, multiple regions of interest

## Abstract

Automated robots are an important part of realizing sustainable food production in smart agriculture. Agricultural robots require a powerful and precise navigation system to be able to perform tasks in the field. Aiming at the problems of complex image background, as well as weed and light interference factors of the visual navigation system in field and greenhouse environments, a Faster-U-net model that retains the advantages of the U-net model feature jump connection is proposed. Based on the U-net model, pruning and optimization were carried out to predict crop ridges. Firstly, a corn dataset was trained to obtain the weight of the corn dataset. Then, the training weight of the obtained corn dataset was used as the pretraining weight for the cucumber, wheat, and tomato datasets, respectively. The three datasets were trained separately. Finally, the navigation line between ridges and the yaw angle of the robot were generated by B-spline curve fitting. The experimental results showed that the parameters of the improved path segmentation model were reduced by 65.86%, and the mPA was 97.39%. The recognition accuracy MIoU of the Faster-U-net model for maize, tomatoes, cucumbers, and wheat was 93.86%, 94.01%, 93.14%, and 89.10%, respectively. The processing speed of the single-core CPU was 22.32 fps/s. The proposed method had strong robustness in predicting rows of different crops. The average angle difference of the navigation line under a ridge environment such as that for corn, tomatoes, cucumbers, or wheat was 0.624°, 0.556°, 0.526°, and 0.999°, respectively. This research can provide technical support and reference for the research and development of intelligent agricultural robot navigation equipment in the field.

## 1. Introduction

Food security has become a hot issue in international agricultural development. In order to further improve production efficiency and economic benefits, smart agriculture and unmanned farms are an inevitable trend in future agricultural development. The use of intelligent agricultural machinery equipment can reduce the workload of farmers. When working in the field, the path navigation of intelligent agricultural machinery is one of the key technologies to realizing precision agriculture [1]. At present, the most popular systems used for the navigation of agricultural machinery are based on global positioning system (GPS) technology and machine vision [2]. Vision sensors are combined with deep-learning algorithms to process farmland image information. They can be used to extract the navigation paths of agricultural robots. Machine vision can be used to prevent or reduce the damage of seedlings during the autonomous navigation of agricultural machinery [3,4]. In the field of agricultural robot navigation technology, visual navigation has become a hot research direction in the field of robot navigation because it has characteristics of wide detection information, complete information acquisition, and low price. However, a navigation scheme based on machine vision still faces the challenges of changing field illumination environment and complex field road conditions. Therefore, environmental information perception is one of the key technologies of an intelligent agricultural robot system that determines the autonomous navigation ability and operational level of agricultural robots.

Machine-vision-based field navigation is sometimes unstable due to long computational response times and is susceptible to changes in the natural environment [5,6,7]. Deep learning has developed rapidly in the field of machine vision in recent years, and semantic segmentation techniques based on deep learning are one of the important methods for the recognition and analysis of complex image scenes. Among them, convolutional neural network algorithms are even more widely used in various agricultural vision task scenarios [8,9,10]. In terms of structured road recognition, convolutional neural networks have been widely used for autonomous driving under structured roads [11]. At the same time, the use of machine vision navigation technology based on deep learning can improve the accuracy and robustness of machine vision algorithms, thus avoiding the limitations caused by the artificial selection of image features [12]. Some scholars have carried out research in related fields of unstructured path recognition and navigation with the help of deep-learning methods. Lin et al. [13] implemented a pixel-level road detection and robot navigation control scheme based on deep learning. Li et al. [14] completed the semantic segmentation of field road scenes in hilly and mountainous areas based on a fully convolutional network (FCN) model, and the mean intersection over union (MIoU) reached 0.732. Based on the DeepLabV3+ framework, Baheti et al. [15] proposed a lightweight segmentation network framework through improvements, and the MIoU reached 0.684. Vijay et al. [16] proposed a SegNet network structure based on FCN, which improved the recognition accuracy of image semantic segmentation for autonomous driving. Han et al. [17] proposed an orchard visual navigation method based on the U-net network aiming at a complex image background and many interference factors that visual navigation systems face in an orchard environment. The optimal MIoU value of the semantic segmentation model could reach 0.895, and the average processing time of a single frame image was 0.154. Vignesh et al. [18] proposed a path recognition method for strawberry crop rows based on a convolutional neural network (CNN) model, and the proposed algorithm provided a solution for robot navigation along strawberry rows.

The application of autonomous navigation technology in weeding is the current development direction of precision agriculture. However, so far, the prevention and control of field weeds are mainly based on manual operations, which are inefficient. For the postprocessing process of robot navigation line extraction, in recent years, many scholars have developed machine-vision-based crop line and navigation path detection algorithms, among which the Hough transform and its variants, as well as least squares, are the mainstream fitting methods.

The Hough transform [19] is one of the most widely used line-fitting methods and is particularly robust when dealing with discontinuous crop rows or fields with many weeds. Many applications of Hough-transform-based crop row recognition in automatic navigation can be found in the literature [5,20,21,22]. Least squares is a mathematical optimization technique that finds the best functional match to the data, minimizing the sum of squared deviations to fit a robot’s navigation path line. For example, Zhang et al. [7], Song et al. [23], and Zhou et al. [24] used the least squares method or its variants to fit maize crop rows and used them in a visual navigation system for robots.

Although the Hough transform and the least squares method have many advantages, when crop ridges have curved transformation, it may cause the problem of failure of navigation line extraction. According to the above methods, this research analyzes and condenses a crop ridge detection method based on selecting the driving area as the area of interest. Zhou et al. [24] selected the drivable area of interest for a plant protection robot with traditional image-processing methods, but the applicability for multicrop environments, such as tomatoes, cucumbers, and wheat, is still unknown. Aiming at this problem, an adaptive multiple regions of interest agricultural robot navigation path recognition method based on deep-learning semantic segmentation is proposed. The algorithm has strong adaptability in different field environments, including different light intensities, weed pressures, and camera positions. The extracted navigation lines can meet the precise requirements of robot navigation. The calculation response time of the algorithm can be controlled within a reasonable time range and has good real-time performance.

At present, the following problems still exist in the field ridge path recognition method based on semantic segmentation. Firstly, most of the existing research has focused on robot navigation path recognition in a single crop ridge scene, lacking a relatively general method; secondly, the semantic segmentation algorithm based on deep learning can improve the computational accuracy, but at the same time, it also needs to take into account the balance between the computing power requirements of computing resources.

In response to the above problems, based on the existing research results, it is found that U-net is a network structure using U-shaped encoding–decoding [25]. It is suitable for image segmentation tasks with simple semantics, large gray-scale ranges, and unclear boundaries [17]. Therefore, a real-time semantic segmentation model of corn ridge images based on U-net is established based on a convolutional neural network. U-net is a lightweight network. It has the advantages of less computer memory data, fast image-processing speed, and strong portability. In order to speed up the network operation and improve the real-time performance of path recognition, on the basis of U-net, we propose the Faster-U-net deep-learning model for the recognition and prediction of corn crop ridges. Through small sample transfer learning, the prediction performance of the Faster-U-net model is verified using three datasets, including cucumbers, tomatoes, and wheat. According to the mask information of the navigation path generated by the model, the navigation information is extracted, the fitting midpoint is generated, and finally multisegment cubic B-spline curve fitting to extract the navigation line of the robot is performed to solve the problem of poor generalization of the recognition of the navigation path between single crop ridges.

## 2. Materials and Methods

**Figure 1:**
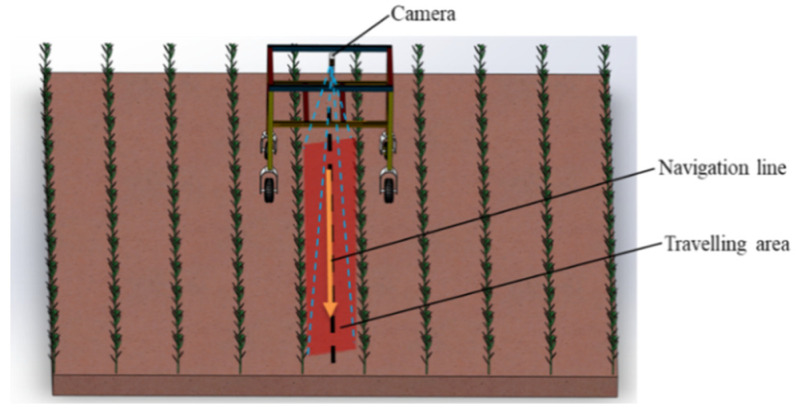
Navigation of agricultural robot in fields.

**Figure 2:**
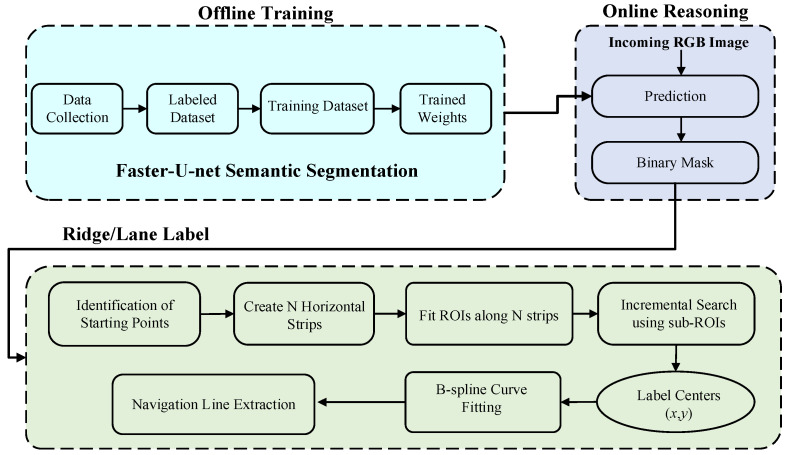
Pipeline for the proposed adaptive multi-ROI-based image semantic segmentation is presented as three subparts: offline training involved data collection and training the dataset, offline reasoning made a prediction over an incoming RGB image, and navigation line extraction applied the fitting procedure over the predicted mask with the proposed adaptive multi-ROI method.

The navigation path recognition method between crop ridges proposed in this paper consisted of two parts: road semantic segmentation (offline training and offline reasoning) and navigation line extraction. The road semantic segmentation was based on the proposed Faster-U-net fully convolutional neural network to perform pixel-level recognition of information between ridges in crop images and obtain the corresponding mask regions. According to the semantic segmentation results, the B-spline curve-fitting method was used to extract the navigation path lines between the ridges of the robot. The key insight of the method presented in this study was to extract the traveling area as the region of interest (ROI). The navigation line was extracted in the ROI, as shown in Figure 1, and the flow chart of the entire navigation line extraction is presented in Figure 2.

### 2.1. Image Acquisition and Dataset Construction

In this study based on an actual robot working scene in the field, four crops were selected to represent common crop ridge navigation scenes based on crop growth posture. Relatively low common crops were represented by cucumbers and wheat at the seedling stage; medium-height crops were represented by maize at mid-growing; and relatively tall crops were represented by tomato plants at maturity. 

#### 2.1.1. Data Sources and Labeling

**Figure 3:**
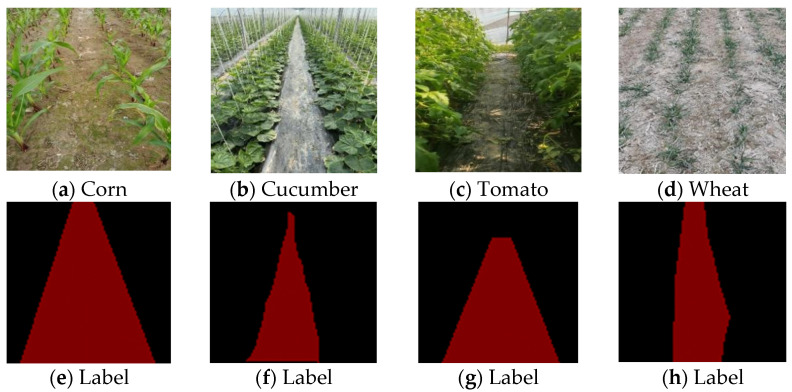
Different crop datasets and labeling results.

Lens model M1224-MPW2 of a Daheng camera was chosen as the vision sensor for this paper. When collecting, the vision sensor was installed on the mobile platform of a robot, and current crop images between the ridges were continuously collected when the mobile platform moved. In May 2021, the image data of tomato ridge rows at the mature stage were collected in a tomato-planting experimental field in the Bei chen District, Tianjin. In August 2021, the image data of corn plants at the jointing stage were collected in the Lingcheng District, Dezhou City, Shandong Province. In March 2022, the image data of cucumbers at the seedling stage were collected at a vegetable greenhouse planting base in Xin County, Liaocheng City, Shandong Province. By collecting images of crop rows under different weather conditions, different environmental backgrounds, and different light intensities, the characteristics of realistic field scenes in natural environments can be better highlighted, thereby improving the diversity and generality of the sample data. Then, 2000 images of the navigation path between corn ridges with a resolution of 1900 × 1180 were randomly selected from the collected data and annotated to generate a corn dataset; 1000 images of cucumbers between ridges, 1000 images of wheat between ridges, and 1000 images tomato ridges with the same resolutions were collected to generate the cucumber dataset, the wheat dataset and the tomato dataset, respectively. An example image is shown in Figure 3.

In this paper, the visual navigation path semantic segmentation model between crop ridges belonged to the category of supervised learning, and the manually annotated semantic images were used as training samples. Because the collected images had no semantic information or labels, Labelme software was used to annotate the image data of the navigation paths between crop ridges. It stored the annotated files in json format, and then converted the annotated files to png-format labeled images through batch conversion. According to the research goal, this paper only labeled the navigation path pixels between crop ridges. The moving path of the mobile platform between the crop ridges was marked in red, and the rest was shown in black as the background. The coordinate information of the edge of the crop row in the labeled file was read, and a gray-scale map of the mask area corresponding to each image was obtained, as shown in Figure 3. As shown in Table 1, the original image size was scaled to 512 × 512 to consider the computational complexity of the semantic segmentation algorithm and the occupation of memory resources. The navigation path data set between corn ridges was divided into a training set and a validation set according to a 4:1 ratio, with a total of 1600 images in the training set and 400 images in the validation set. For the three datasets of cucumbers, wheat, and tomatoes, we set 800 images from each dataset as the training set and 200 images as the validation set. 

#### 2.1.2. Data Enhancement

Because the acquired image data were difficult to completely cover all the environments, there was an uneven distribution of the number of samples of different categories. To expand the dataset, further increase the diversity of the dataset, enhance the generalization ability and robustness of the model, and achieve data-enhanced small sample learning, in this paper, color transformation (random weakness or enhancement of the luminance of the original input image; random enhancement of the image contrast; dynamic change in chromaticity by 30%) and geometric transformation (image translation; scaling; random rotation by 0°, 90°, 180°, or 270°) were used in the model-training process to enhance the robustness and accuracy of the model. Randomly rotated and flipped images provided directional confirmation of navigation paths between the various crops in the datasets. Random adjustment of the image HSV provided for the adaptation of the model to uneven natural lighting conditions. At each iteration of training, to read the image data, the original image performed the above steps in sequence. Each step had a 50% probability of triggering a change. This was a 50% chance that it received each of the transforms.

#### 2.1.3. Evaluation Basis for Ridge Semantic Segmentation

In the process of semantic segmentation, the model inference speed and accuracy were used as evaluation quantitative indicators. The accuracy estimation of the model was represented by the average intersection ratio, and the inference speed represented the real-time level of the model. The model inference speed was represented by the number of frames processed per second. This paper excluded all dataset reading times and image-loading times when calculating the model inference speed and only calculated the time from the input in the model to the output of the image.

Average pixel accuracy MPA (mean pixel accuracy) is the ratio of the number of correctly predicted pixels for each category to the number of all pixels in this category. We then calculated the average values of all the categories. The formula was:

(1) $$MPA=\frac{1}{k+1}\sum_{i=0}^{k}\frac{p_{ii}}{\sum_{j=0}^{k}p_{ij}}$$

MIoU is a standard metric in image semantic segmentation [1], which calculates the ratio of the intersection and union of two sets, such as the true value and the predicted value. The IoU value was calculated for each category and then averaged, and the formula was as follows:

(2) $$MIoU=\frac{1}{k+1}\sum_{i=0}^{k}\frac{p_{ii}}{\sum_{j=0}^{k}p_{ij}+\sum_{j=0}^{k}p_{ji}-p_{ii}}$$

In the formula, *p_ii_* represents the number of true values *i* and is predicted to be *i*, which is the number of true values; *p_ij_* represents the number of true values *i* but is predicted to be *j*, which is the number of false positives; *p_ji_* represents the number of true values *j* but is predicted to be *i*, which is the number of false negatives; and *k* + 1 represents the number of categories containing empty classes. Since the objects in this study were only for the navigation path and image background, the value of *k* here was taken as 1.

### 2.2. Path Recognition Model between Crop Ridges

Based on deep learning and a semantic segmentation algorithm, crop ridge images were classified at the pixel level. The input image was encoded by the convolution layer and pooling layer when it was processed by the deep neural network. The output of the encoder was decoded by the decoder to generate the output image. The decoder was generated by up-sampling through bilinear difference or deconvolution for up-sampling, increasing the resolution of higher-order features and ensuring that the output image size was the same as the input image, thus predicting each pixel class.

Thus, predictions were made for each pixel category. The more common network models that can be used for semantic segmentation are Segnet, Deeplab V3+, and U-net, which have the characteristic of high accuracy. However, these four network models have a large amount of computation and relatively slow operation [3]. Because of the problems existing in the above neural network models, this study proposed the Faster-U-net model for real-time accurate segmentation of navigation paths between crop ridges in natural environments based on the U-net model, which was optimized and improved with a deep-learning algorithm based on channel pruning [26,27].

#### 2.2.1. Construction of Semantic Segmentation Model between Crop Ridges

A general semantic segmentation model consists of two parts: an encoder and a decoder. The encoder uses convolution, pooling, linear rectification functions, and other operations to form a feature extraction network and encode the input image features and pixel position information. The decoder uses deconvolution or up-pooling operations to map low-resolution features of the encoder output to a high-resolution pixel space to obtain a dense pixel prediction classification.

**Figure 4:**
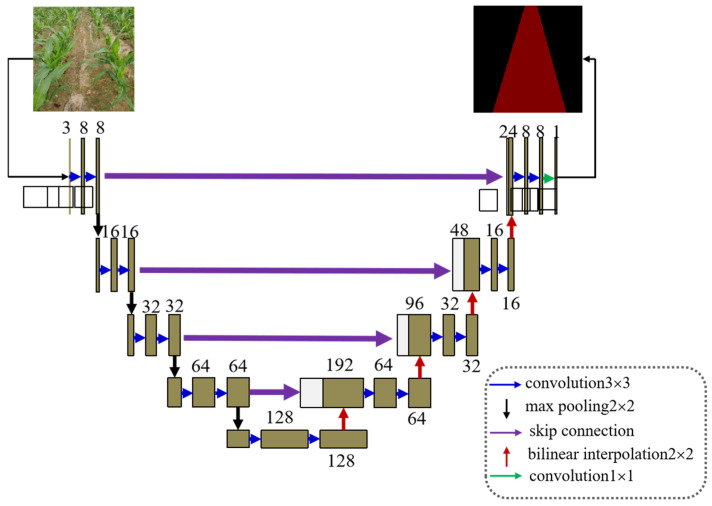
Faster-U-net network model structure.

The overall structure of the U-net network is in the shape of a “U”, and the network structure is completely symmetrical. The feature map is dimensionally spliced by skip connection, which can retain more position and feature information. The segmentation performance is better than other network structures on small-sample datasets, and it is suitable for the semantic segmentation tasks of small-sized objects [17,28]. To satisfy the real-time requirements of weeding robot navigation path recognition, the U-net structure was optimized in this paper. Because the classification task of a weeding robot in the agricultural scene navigating between rows of crops is relatively simple [3], the number of convolution kernels and the number of feature maps in the forward-pass derivation process were reduced by 70%. The reduction in the parameters of the Faster-U-net model provided a guarantee for the real-time performance of path recognition. The network architecture of Faster-U-net is shown in Figure 4.

Although U-net is a lightweight convolutional neural network, because our path segmentation was relatively simple, it could reduce computational consumption in terms of computation. In order to further reduce the complexity and parameters of the model under the condition that the cutting accuracy level was basically unchanged, in this paper, the deconvolution up-sampling operation commonly used in U-net was replaced with bilinear interpolation during the research process. Considering deployment on embedded devices with weak computing power in the future, further pruning of the network model was a necessary method. Channel pruning is eliminating unimportant channels and their associated input and output relationships by identifying the channels of the network. Compared with a layer-pruning algorithm, this algorithm can reduce the number of parameters that need to be stored [26]. It has lower hardware requirements. Therefore, it is convenient to deploy on small computing platforms, such as embedded devices and mobile terminals. 

**Figure 5:**
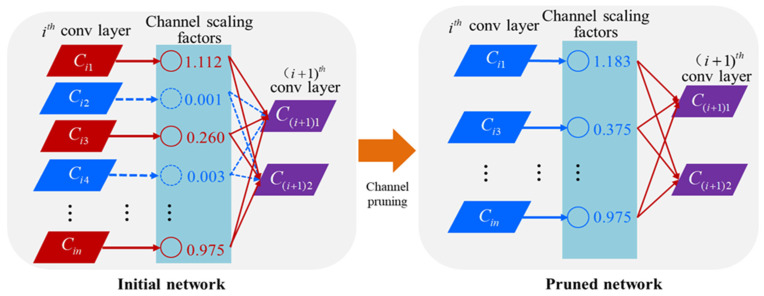
Schematic diagram of the principle of channel pruning based on the U-net model.

As shown in Figure 5, in the channel-pruning algorithm, the gamma coefficients of the batch normalization (BN) layer were used to evaluate the contribution scores of the input channels. According to the distribution of the gamma coefficients and the pruning rate of the channel-pruning algorithm, the channel with the large contribution (red solid line) was retained, and the channel with the small contribution (blue dotted line) was deleted. The neurons contributing to lower channels did not participate in the connection. In this study, the specific steps of channel pruning included the following aspects:

(1) Sparse training by applying the L1 rule constraint to the batch normalization layer coefficients of the U-net corn ridge row segmentation model so that the model was adjusted in the direction of structural sparseness.
(2) Channel pruning. After the sparse training was completed, channel pruning was performed at a certain pruning rate to generate a simplified model with less storage space.
(3) Local adjustment of the pruned network model to solve the problem of excessive model accuracy loss after channel pruning to effectively restore the lost accuracy.

#### 2.2.2. Transfer Learning

**Figure 6:**
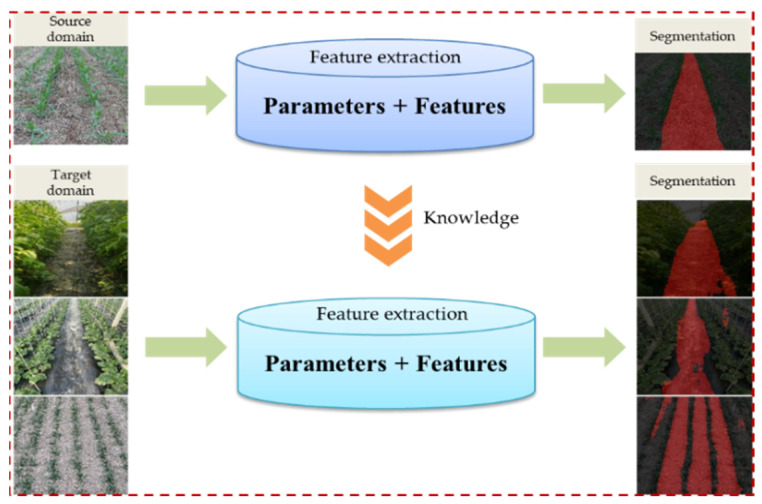
Visual transfer learning.

Transfer learning is a process in which a deep-learning model uses existing knowledge to learn new knowledge, and its core is to find the similarity between existing knowledge and new knowledge [29]. The existing knowledge in the transfer learning process is used as the source domain. As shown in Figure 6, the source domain in this research work was the feature space of the navigation path recognition dataset between corn ridges. The target domain was the feature space of the navigation path dataset between rows of crops, such as cucumbers, tomatoes, and wheat. The source domain data and the target domain data were different but related. A learning algorithm based on the generative model was introduced here, and the joint probability P(X,Y) was calculated first, and then P(Y|X) was calculated. In this way, the generative model provided a good mechanism. The different distributions of the source domain and target domain data could be modeled, and the knowledge transfer between the source domain and the target domain could be realized to improve the performance of the algorithm [30]. 

Model transfer learning is based on shared parameters. Under the premise that the source domain data and the target domain data could share some model parameters, the model learned from the feature space of the source domain was applied to the feature space of the target domain. The new model was obtained according to the target domain learning. 

#### 2.2.3. Navigation Line Extraction

**Figure 7:**
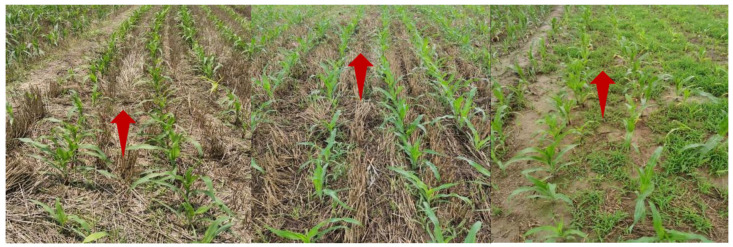
Rows of corn with varying degrees of curvature. The left image is a curved corn row in bright light. The middle image is a curved corn row in normal light. The final image is a curved corn row on a cloudy day.

After the training of the semantic segmentation model was completed, the regression probability of each pixel could be obtained by inputting a test image. After the prediction results of the model were binarized, the navigation prediction path of the crop row robot could be generated. At present, most of the methods for identifying the navigation path of agricultural robots use the Hough transform, least squares method, and variants of straight-line fitting for linear fitting, which are suitable for straight-line paths. In a corn field environment, because the agricultural machinery does not sow in a straight line. The sowing path is always curved. At this point, the path shows a curved change (Figure 7). 

**Figure 8:**
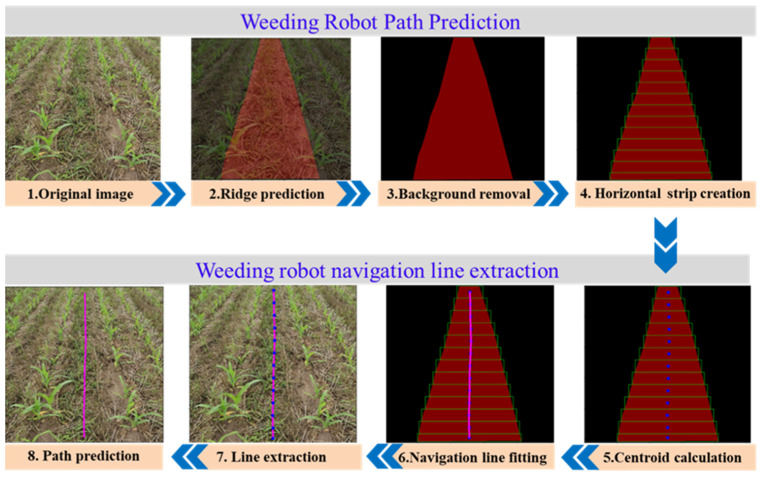
Main procedures of this paper.

If the method of straight-line fitting is used again, failure may occur or the error of straight-line fitting may become larger. To avoid the problem, this paper used the method of creating N horizontal strips based on the video frame, fitting the ROI along the acquired horizontal strip, and calculating the center point of each sub-ROI. Compared with polynomial, exponential, and logarithmic curves, the B-spline curve has the characteristics of local controllability and continuity because there are irregular fields and curved ridges in farmland ridges. In this paper, the starting point of the navigation, the center point of the ridge line, and the end point of the ridge line were taken as the control points of the B-spline, and multisegment cubic B-spline curve fitting was performed to generate a relatively smooth robot visual navigation line. As shown in Figure 8, the overall technical visualization flow chart of this paper is exemplified.

The navigation line extraction of the intelligent weeding robot was based on n + 1 control vertices *P_i_* (I = 0, 1,..., n), and each time 4 adjacent control vertices were taken out, a cubic B-spline curve could be obtained, wherein the expression of the cubic B-spline curve of the *i*-th segment was:

(3) $$\left\{\begin{array}{l} C_{i,4}(t)=\sum_{j=1}^{4}N_{j,4}(t)P_{i+j-2}(t\in [0,1])\\ N_{j,4}(t)=[N_{1,4}(t),N_{2,4}(t),N_{3,4}(t),N_{4,4}(t)]\\ N_{1,4}(t)=(-t^{3}-3t^{2}-3t+1)/6\\ N_{2,4}(t)=(-3t^{3}-6t^{2}+4)/6\\ N_{3,4}(t)=(-3t^{3}-3t^{2}+3t+1)/6\\ N_{4,4}(t)=t^{3}/6 \end{array}\right.$$

In the formula, *t* represents the curve parameter, and *N_j_*_,4_(*t*) represents the basis function of the spline curve.

By substituting the basis function into the matrix, the expression corresponding to the cubic B-spline curve of the *i*-th segment could be obtained:

(4) $$C_{i,4}(t)=\frac{1}{6}{\left[\begin{array}{l} t^{3}\\ t^{2}\\ t\\ 1 \end{array}\right]}^{T}\left[\begin{array}{cccc} -1 & 3 & -3 & 1\\ 3 & -6 & 3 & 0\\ -3 & 0 & 3 & 0\\ 1 & 4 & 1 & 0 \end{array}\right]\left[\begin{array}{c} P_{i-1}\\ P_{i}\\ P_{i+1}\\ P_{i+2} \end{array}\right]$$

The coordinate positions, first-order derivatives, and second-order derivatives of the start and end points of the *i*-th B-spline curve were:

(5) $$\left\{\begin{array}{l} C_{i,4}(0)=(P_{i-1}+4P_{i}+P_{i})/6\\ C_{1,4}(1)=(P_{i}+4P_{i+1}+P_{i+2})/6 \end{array}\right.$$

(6) $$\left\{\begin{array}{l} C_{i,4}^{\prime }(0)=(P_{i+1}-P_{i-1})/2\\ C_{i,4}^{\prime }(1)=(P_{i+2}-P_{i})/2 \end{array}\right.$$

(7) $$\left\{\begin{array}{l} C_{i,4}^{\prime \prime }(0)=P_{i-1}-2P+P_{i+1}\\ C_{i,4}^{\prime \prime }(1)=P_{i}-2P_{i+1}+P_{i+2} \end{array}\right.$$

Based on the above derivation, a multisegment cubic B-spline curve was fitted on the center point of the path, fitting to generate the visual navigation line of the weeding robot. Based on the spline-curve-fitting principle, the smaller the height of the rectangular frame, the greater the number of fitting center points, and the accuracy of the navigation line extraction also changes accordingly. If the threshold value of the scanning rectangle is selected as too large, the number of center points formed is reduced, resulting in a larger error in the fitting of the navigation line. If the threshold value of the scanning rectangular frame is selected to be smaller, the number of center points formed is larger, which is prone to over-fitting of the navigation line.

**Figure 9:**
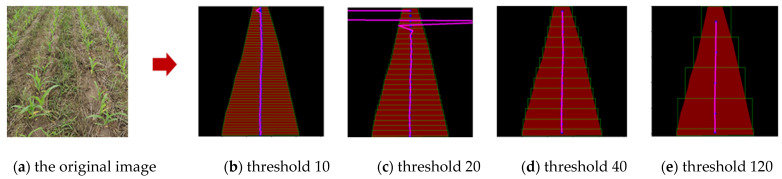
Incremental sub-ROI search analysis.

After the crop ridge prediction was completed, horizontal bands were created on the generated binary mask image to form the incremental search box of the sub-ROI. As shown in Figure 9, compared with the test results, when the threshold of the scanning rectangle frame was set to 40 pixels, the extracted navigation line could effectively reflect the path information of the crop ridges. The overall curve was smooth, and the calculated amount was relatively low. Therefore, the height of the rectangular frame was selected to be 40 pixels for research in the later stage.

## 3. Results and Discussion

### 3.1. Test Platform

The test platform was a MACHENIKE computer. The computer processor used for model training in the experiment was an Intel(R) Xeon(R) Gold6147M. The reference frequency was 2.50 ghz, with 16 GB memory, 1024 GB solid-state drive, an NVIDIA RTX 3060 graphics processing unit (GPU), 8 GB video memory, and a Windows 10 operating system. Based on Python 3.8 language, the program was carried out under PyTorch deep-learning framework. CUDA 10 was selected for unified computing device architecture. The accelerated library version of the neural network was CUDNN V10.0.

### 3.2. Image Semantic Segmentation between Crop Ridges

In order to verify the accuracy, stability, and robustness of the path recognition method proposed in this paper in the field dataset, path segmentation and navigation line extraction experiments were carried out, respectively.

#### 3.2.1. Path Segmentation Evaluation Index

The robot drove between the rows of crops. The semantic segmentation of the path was mainly based on the accuracy rate and the intersection ratio. The formulas were:

(8) $$P=\frac{T_{P}}{T_{P}+F_{P}}\times 100\%$$

(9) $$I=\frac{T_{P}}{T_{P}+F_{P}+F_{N}}\times 100\%$$

where *P* represents the category pixel accuracy for the semantic segmentation. *I* represents the intersection over union. *T*_*P*_ represents the number of positive sample pixels determined as positive samples; *F*_*P*_ represents the number of pixels of negative samples determined as positive samples; *F*_*N*_ represents the number of positive sample pixels determined as negative samples.

#### 3.2.2. Network Model Training

The hyperparameters used in the training of the navigation path recognition between crop ridges in this paper were as follows: the number of iterations (epoch) was 100 times. The navigation path recognition task between ridges was clear, which was quite different from the conventional semantic segmentation object recognition task. At the same time, the corn navigation path data were sufficient, so the corn dataset did not use transfer learning in training. When performing transfer-learning training on the cucumber, wheat and tomato datasets, the optimal model parameters obtained by direct training on maize datasets were used as the initial training parameters of the new model. The remaining training parameters and learning rate strategy were consistent with the original training.

In the network training, the batch training method was adopted. The training set and the validation set were divided into multiple batches for training, and the traversal calculation of all the training set images in the network model was an iteration. The network model initialization adopted the loading of pretrained weights to initialize the backbone network. The initial learning rate was 0.001, and the Adam algorithm was used to optimize and calculate the adaptive learning rate of each weight parameter.

BCEDiceLoss was used as the training loss function. This loss function is weighted by dice loss and binary cross-entropy loss (BCELoss) weighted by weight, and the calculation formula was:

(10) BCEDiceLoss=w×BCELoss+DiceLoss

(11) $$BCEL\mathrm{oss}(y,\hat{y})=-\frac{1}{m}\sum_{i=1}^{m}\left[y_{i}\ln(\frac{1}{1+e^{-\hat{y_{i}}}})+(1+y_{i})\ln(1-\frac{1}{1+e^{-\hat{{\overset{\cap }{y}}_{i}}}})\right]$$

(12) $$DiceLoss(Y,\hat{Y})=1-\frac{2\left|Y\cap \hat{Y}\right|+Smooth}{\left|Y\right|+\left|\hat{Y}\right|+Smooth}$$

where *w* is the weight of BCELoss, $\overset{\wedge }{y}$ is the image pixel value in the label graph, $\overset{\cap }{y}$ is the image pixel value in the prediction graph, and *m* is the pixel value in each image. *Y* is the label graph, and $\overset{\cap }{Y}$ is the prediction graph; Smooth is to avoid a situation where the denominator is 0, and its value is 1 × 10^−5^.

**Figure 10:**
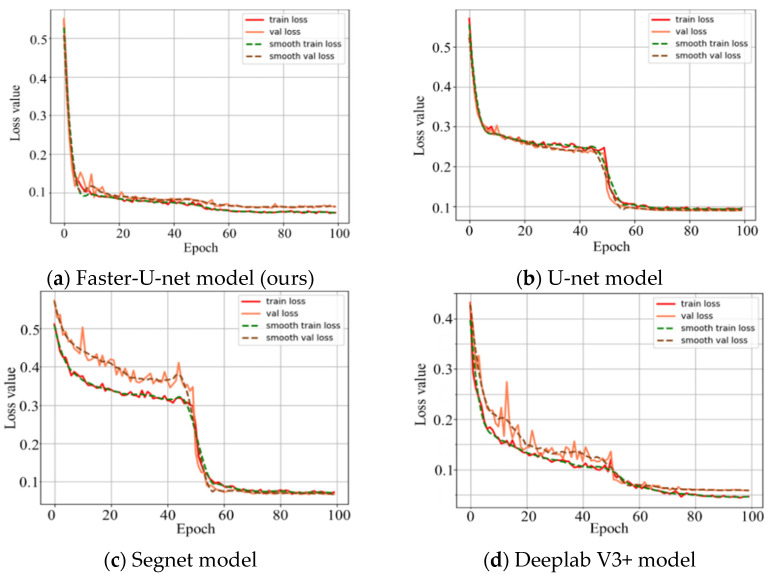
Model training loss function curves. (**a**) The loss function curve obtained based on the Faster-U-net model. (**b**) The loss function curve obtained based on the U-net model. (**c**) The loss function curve obtained based on the Segnet model. (**d**) The loss function curve obtained based on the Deeplab V3+ model.

DiceLoss judges the classification effect of a prediction map from the global perspective, and BCELoss judges the classification effect of each pixel from the details. In this paper, the identification of the navigation path between crop ridges needed to pay attention to the accuracy of global judgment and take into account the details, so DiceLoss was given a higher weight in the calculation of BCEDiceLoss. In this paper, n = 0.5. In the training process, the loss function curves of the Deeplab V3+, Segnet, U-net, and Faster-U-net models are shown in Figure 10.

**Figure 11:**
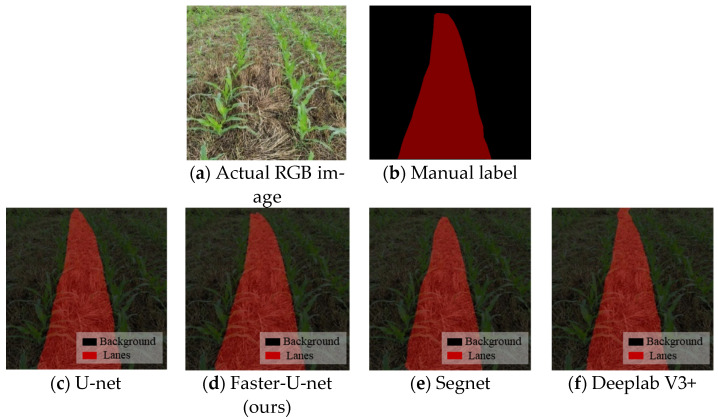
Prediction results of corn navigation path. (**a**) Actual RGB image was collected in the field. (**b**) Manual label gave path semantic information. (**c**) The path result as predicted by the U-net network. (**d**) The path result as predicted by the Faster-U-net network. (**e**) The path result as predicted by the Segnet network. (**f**) The path result as predicted by the Deeplab V3+ network.

It can be seen from Figure 10 that the loss function of the training set gradually decreased with the increase in the number of iterations. Further, the prediction results of the Faster-U-net, U-Net, Segnet, and Deeplab V3+ models were visualized and compared, respectively, and the results shown in Figure 11 were obtained by taking the recognition of navigation paths between ridges in corn fields as an example.

Without distinguishing the factors affected by lighting, the U-net, Segnet, Deeplab V3+, and other models were selected for comparison and testing with the model in this paper. The performance of the model was evaluated by indicators such as average pixel accuracy, average area coincidence, detection speed, and number of parameters. The above models were trained based on the maize ridge dataset, and the relevant metrics were calculated on the test set. Table 2 compares the performance parameters of different network models.

Compared with three network models, such as U-net, Segnet, and Deeplab V3+, the average pixel accuracy predicted by our model reached 97.39%. The MIoU value was 93.86%. Among the four models, the Faster-U-net network predicted the highest path accuracy. The speed of image processing reached 22.32 fps/s. The number of parameters was 2.68 × 10^6^. The prediction path accuracy of the U-net network model was no different from the Faster-U-net prediction value. However, in terms of the other three indicators, the performance parameters of the network model we extracted were relatively good. Although the Segnet model is a classic lightweight neural network, the performance parameters were all lower than Faster-U-net. The Deeplab V3+ is a heavyweight network model with the largest number of parameters. The Deeplab V3+ was the lowest in detection real-time. From the analysis, our network model had good application prospects in the field of agricultural robot visual navigation.

#### 3.2.3. Segmentation Accuracy Test

**Figure 12:**
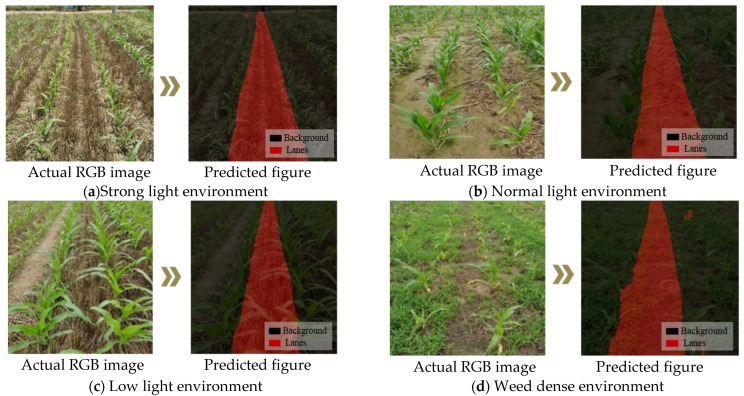
Prediction results of Faster-U-net network model in complex environment. (**a**) The path as predicted by agricultural robot in strong light environment. (**b**) The path as predicted by agricultural robot in normal light environment. (**c**) The path as predicted by agricultural robot in low light environment. (**d**) The path as predicted by agricultural robot in weed-dense environment.

Considering that the light environment is complex and changeable when a robot is working in the field, it can be basically divided into three types of environments: strong light, ordinary light, and weak light (Figure 12). Based on the Faster-U-net network model, this study explored the influence factor of lighting on crop ridge path segmentation in the above three environments.

As shown in Figure 12, the network model based on Faster-U-net could correctly identify the ridges between crop rows, thus laying a foundation for the later extraction of navigation lines. Crop row images were acquired, and a mask was generated to predict the crop ridge. This mask was used to perform the navigation. The time consumption was 15 ms for a single-frame image binary mask. As shown in Table 3, the model segmentation accuracy results of the model under three conditions of weak light, normal light, and strong light were tested respectively.

It can be seen from Table 2 that the proposed model could better overcome the interference of light factors for crop ridge prediction, and the MIoU input results all exceeded 93.70%, while the mPA could reach 97%. The model accuracy performances of the tomato, cucumber, and wheat datasets after transfer learning of the U-net model are shown in Table 4.

For the tomato, cucumber, and wheat navigation path datasets, the MIoU values of the model trained with the transfer-learning method were better than that of the model trained by non-transfer learning. After transfer learning, the MIoU input results of the proposed model and the U-net model were less different. However, in the scenario of a tomato ridge navigation path with low identification difficulty, the network model proposed in this paper achieved a higher MIoU values in both transfer learning and non-transfer learning, which were increased by 0.0039 and 0.2901, respectively, compared with U-net before improvement. The transfer learning consequences for both cucumbers and wheat were better than the non-transfer learning consequences. From the results of the whole data training, Faster-U-net achieved better learning results than U-net, Segnet, and Deeplab V3+. This indicates that Faster-U-net was more efficient in path recognition.

### 3.3. Navigation Line Extraction Test

**Figure 13:**
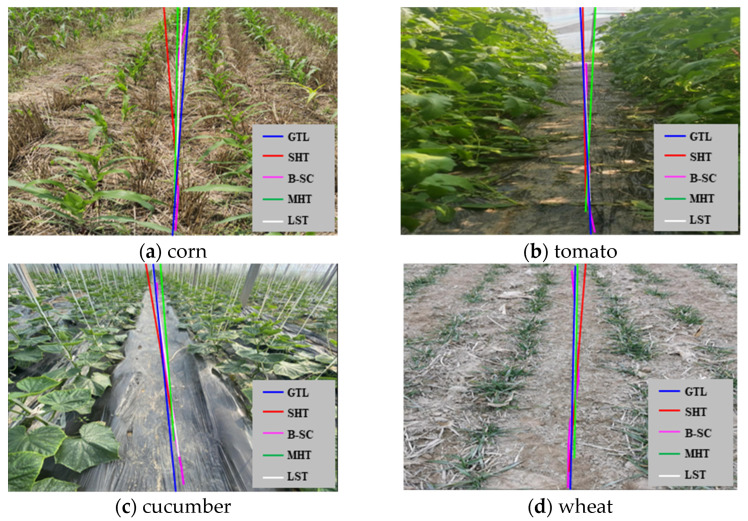
Comparison of line fittings. GTL stands for the ground-truth line. The ground-truth line indicates the line of evaluation. SHT stands for standard Hough transform. B-SC stands for B-spline curve. MHT stands for median-point Hough transform. LST stands for least squares method.

The network model based on Faster-U-net predicted the results of crop ridges and then extracted the navigation line of the weeding robot. Comparative experiments were carried out on maize, tomatoes, cucumbers, wheat, and four other crops using standard Hough transformation, B-spline curve fitting, median Hough transformation, least squares linear regression, and other line-fitting methods, as shown in Figure 13. In the figure, the red lines represent the fitting results of the standard Hough transform, the pink lines represent the fitting results of B-spline, the green lines represent the fitting results of the median-point Hough transform, and the white lines represent the fitting results of the least squares method.

In order to calculate the yaw angle of the robot to extract the navigation line, the center line of the generated path was directly selected. The center line was taken as the benchmark to quantify the angle difference, and the center line was regarded as the idealized robot navigation line. It was compared with the least squares method, B-spline curve, standard Hough transform, median Hough transform, and other methods for yaw angle difference. The navigation line extraction results of corn, cucumbers, tomatoes, and wheat showed the average difference in the yaw angle, as shown in Table 5.

It can be seen from Table 4 that the average difference in the yaw angle between the B-spline curve, least squares, and median-point Hough transform methods was small, and the extraction accuracy of the navigation line was similar to the MIoU results of the navigation path. However, the average angle differences between the standard Hough transform line detection and the yaw angle of the B-spline curve in the maize, cucumber, tomato, and wheat fields were 7.267°, 5.153°, 2.422°, and 0.033°, respectively.

**Figure 14:**
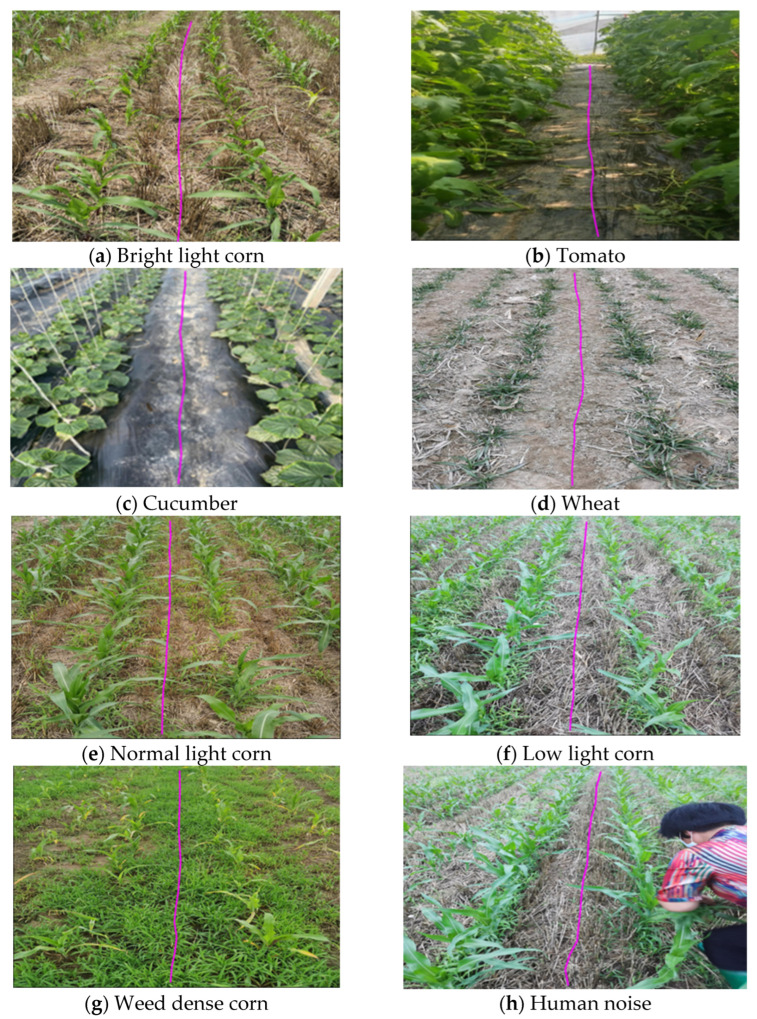
Extraction results of navigation line based on B-spline curve.

Based on the above discussion and analysis, due to the tortuous path of sowing crops in the field, the ridges of the crops are correspondingly tortuous. The predicted path of the field-weeding robot has a curved problem. If the navigation line is generated using the least squares method [24], the standard Hough transform [31], or the median-point Hough transform [21], the above three methods belong to the line-fitting detection method. Using a method of straight-line detection and fitting can cause the agricultural robot to damage and press the seedlings, which violates the robot’s intention of correct visual navigation. Therefore, we chose the B-spline curve to fit the navigation reference point to complete the extraction of the robot’s navigation line. In Figure 14, it shows the situation of robot navigation line extraction in a complex and changeable environment.

Based on the above, ridges under multiple environmental variables can better identify and extract a robot’s navigation line. Therefore, the Faster-U-net model proposed in this paper could accurately identify the navigation paths of other categories of crops under the condition of transfer learning. The average yaw angles of the robot’s navigation line in corn, cucumber, tomato, and wheat crops were 0.624°, 0.556°, 0.526°, and 0.999° based on the predicted ridge line extraction through B-spline curve. The yaw angle difference in the extracted navigation lines was small, and the time for extracting navigation based on the B-spline curve was 0.001 s, which could meet the robot’s navigation accuracy requirements and real-time performance. Therefore, under the current crop row environments and hardware configuration, the proposed path identification method had better navigation accuracy.

## 4. Conclusions

This study addressed the problem of the vision-based navigation operation of intelligent mid-tillage weeding robots in fields and greenhouses between field monopolies. The Faster-U-net semantic segmentation model was proposed based on the semantic segmentation algorithm U-net through channel pruning and optimization of the model structure. Through transfer learning, the effective extraction of ridge navigation lines of four crops, including maize, tomatoes, cucumbers, and wheat, was realized under the complex environment of natural lighting and crop leaf shading in the field. The main conclusions were as follows:

(1) This research modified U-net and proposed the Faster-U-net semantic segmentation model. The recognition accuracy MIoU values of the model for corn, tomatoes, cucumbers, and wheat were 93.86%, 94.01%, 93.14%, and 89.10%, respectively. Under the premise of ensuring accuracy, the processing speed of the single-core CPU was 22.32 fps/s, which could basically meet the real-time requirements of robots in embedded environments.

(2) The navigation path of the weeding robot was predicted based on the Faster-U-net semantic segmentation model. Based on the segmentation of crop ridges by visual navigation path, a sub-ROI incremental search method was proposed to obtain datapoints for inter-monopoly navigation combined with B-spline curves to extract the navigation lines of the robot. The average angle differences in four types of environments, including corn, tomatoes, cucumbers, and wheat, were 0.624°, 0.556°, 0.526°, and 0.999°, respectively. It had good adaptability to the shape of the road mask in different viewing angles and different crop environments.

In this work, we proposed a navigation line extraction system with machine vision using the semantic segmentation of deep learning combined with adaptive multi-ROI-based midpoint fitting. What is more, the proposed B-spline curve-fitting procedure applied multiple smaller windows along equally divided horizontal strips in the input image. The tests showcased improved results compared with other fitting methods. Our future work involves searching for the automatic transition when a robot reaches one end of the crop ridges. Therefore, the agricultural robot makes closed-loop field testing for the whole field.

## Figures and Tables

**Table 1:** Dataset settings.

Kind of Dataset	Number of Images	Original Image Resolution	Resized Image Resolution	Training Set	Validation Set
Corn	2000	1900 × 1180	512 × 512	1600	400
Cucumber	1000	1900 × 1180	512 × 512	800	200
Tomato	1000	1900 × 1180	512 × 512	800	200
Wheat	1000	1900 × 1180	512 × 512	800	200

**Table 2:** Performance comparison of different network models.

Network Model	Average Pixel Accuracy/%	MIoU/%	Detection Speed/fps·s^−1^	Parameter Quantity/ × 10^6^
U-net	93.98	92.58	14.45	7.85
Faster-U-net (ours)	97.39	93.86	22.32	2.68
Segnet	94.43	91.75	16.68	29.94
Deeplab V3+	96.16	93.60	12.67	34.67

**Table 3:** Semantic segmentation accuracy test results.

Experimental Conditions	Crop Category	mPA/%	MIoU/%	mP/%
Low light intensity	Corn	97.35	94.30	96.76
Normal light intensity	Corn	97.68	94.92	97.10
Strong light intensity	Corn	97.17	93.70	96.32

**Table 4:** Faster-U-net transfer learning MIoU results comparison.

Crop Category	U-net	Faster-U-net	Segnet	Deeplab V3+
Tomato (transfer learning)	0.9362	0.9401	0.9361	0.9368
Tomato (no transfer learning)	0.6095	0.8996	0.4683	0.2795
Cucumber (transfer learning)	0.8897	0.9314	0.8897	0.8876
Cucumber (no transfer learning)	0.8056	0.8187	0.5673	0.7614
Wheat (transfer learning)	0.8824	0.8910	0.8612	0.8873
Wheat (no transfer learning)	0.6947	0.7675	0.5034	0.6441

**Table 5:** Yaw angle statistics under different crop conditions based on the proposed model. LST stands for least squares method. B-SC stands for B-spline curve. SHT stands for standard Hough transform. MHT stands for median-point Hough transform.

Method	Average Difference in Corn Yaw Angle (°)	Average Difference in Cucumber Yaw Angle (°)	Average Difference in Tomato Yaw Angle (°)	Average Difference in Wheat Yaw Angle (°)
LST	0.638	0.572	0.541	0.996
B-SC	0.624	0.556	0.526	0.999
SHT	7.891	5.709	2.948	1.066
MHT	0.605	0.537	0.546	1.032

## Data Availability

Not applicable.
